# Combining 2-deoxy-D-glucose with fenofibrate leads to tumor cell death mediated by simultaneous induction of energy and ER stress

**DOI:** 10.18632/oncotarget.9263

**Published:** 2016-05-10

**Authors:** Huaping Liu, Metin Kurtoglu, Clara Lucia León-Annicchiarico, Cristina Munoz-Pinedo, Julio Barredo, Guy Leclerc, Jaime Merchan, Xiongfei Liu, Theodore J. Lampidis

**Affiliations:** ^1^ Department of Cell Biology, University of Miami, Miller School of Medicine and Sylvester Comprehensive Cancer Center, Miami, FL, USA; ^2^ National Institutes of Health, Bethesda, MD, USA; ^3^ Cell Death Regulation Group, Bellvitage Biomedical Research Institute (IDIBELL), L'Hospitalet de Llobregat, Barcelona, Spain; ^4^ Department of Pediatrics, University of Miami, Miller School of Medicine and Sylvester Comprehensive Cancer Center, Miami, FL, USA; ^5^ Department of Medicine, University of Miami, Miller School of Medicine and Sylvester Comprehensive Cancer Center, Miami, FL, USA

**Keywords:** 2-deoxy-D-glucose, fenofibrate, energy stress, mTOR, eIF2α

## Abstract

Unregulated growth and replication as well as an abnormal microenvironment, leads to elevated levels of stress which is a common trait of cancer. By inducing both energy and endoplasmic reticulum (ER) stress, 2-Deoxy-glucose (2-DG) is particularly well-suited to take advantage of the therapeutic window that heightened stress in tumors provides. Under hypoxia, blocking glycolysis with 2-DG leads to significant lowering of ATP resulting in energy stress and cell death in numerous carcinoma cell types. In contrast, under normoxia, 2-DG at a low-concentration is not toxic in most carcinomas tested, but induces growth inhibition, which is primarily due to ER stress. Here we find a synergistic toxic effect in several tumor cell lines *in vitro* combining 2-DG with fenofibrate (FF), a drug that has been safely used for over 40 years to lower cholesterol in patients. This combination induces much greater energy stress than either agent alone, as measured by ATP reduction, increased p-AMPK and downregulation of mTOR. Inhibition of mTOR results in blockage of GRP78 a critical component of the unfolded protein response which we speculate leads to greater ER stress as observed by increased p-eIF2α. Moreover, to avoid an insulin response and adsorption by the liver, 2-DG is delivered by slow-release pump yielding significant anti-tumor control when combined with FF. Our results provide promise for developing this combination clinically and others that combine 2-DG with agents that act synergistically to selectively increase energy and ER stress to a level that is toxic to numerous tumor cell types.

## INTRODUCTION

Cancer cells by nature of their uncontrolled growth, proliferation and abnormal micro-environment, are under more stress than normal cells [1]. This inherent trait of tumors presents a therapeutic window that the sugar analog 2-deoxy-D-glucose (2-DG) is particularly well-suited to exploit since it can simultaneously induce different forms of metabolic stress. 2-DG is primarily known as an analog of glucose that causes energy stress through its action as a glycolytic inhibitor. However, as an analog of mannose, 2-DG interferes with n-linked glycosylation resulting in ER stress [2, 3]. Under normoxia these forms and levels of stress induced by a low concentration of 2-DG (< 2 mM) are not enough to be toxic to most carcinoma cell types tested [3, 4]. However, in a number of rhabdomyosarcoma [5] and acute lymphoblastic cell lines [6] grown at normal oxygen levels, 2-DG was reported to induce ER stress-mediated apoptosis. Thus, even under normal oxygen conditions, it appears that a number of non-carcinoma tumor cell types are sensitive to the toxic effects of this dual energy and ER stress inducing sugar analog.

Previously, we reported that multiple myeloma (MM) cells known to be under heightened ER stress, due to the over abundance of antibodies they produce, are selectively sensitive to the toxic effects of various mitochondrial inhibitors at doses that are non-toxic to non-antibody producing B cell leukemias [7]. The rationale for these experiments was based on the understanding that as a function of ER activity or stress, Ca^++^ is released from the ER into the cytoplasm and is attracted to, and re-adsorbed by, the high negatively-charged potential established between the mitochondrial matrix and the outer mitochondrial membrane [8]. Thus, by perturbing mitochondrial function and interfering with Ca^++^ re-entry to the ER, MM cells due to their inherent ER stress would be further stressed and die. Among the many mitochondrial agents that we found to selectively kill MM cell lines was the PPARα agonist, fenofibrate (FF) [7]. This drug has been used safely to lower triglycerides and cholesterol in patients for greater than forty years [9] but in addition to its agonist activity on PPARα, was reported to have inhibitory effects on mitochondrial function [10].

These results led us to the present study in which we speculated whether by inducing ER stress endogenously with 2-DG, carcinoma cells could be made to become “MM-like” in their sensitivity to FF. Moreover, by nature of 2-DG's other activity as a glycolytic inhibitor, combining it with FF would further lower ATP levels adding energy stress. In this report we demonstrate that 2-DG, at a non-toxic low concentration, is synergistic in killing a variety of carcinoma cell types when combined with FF, and that cell death appears to be a result of both energy and ER stress induced by this combination as cells undergo necrosis as well as apoptosis. Our results reveal a heretofore undetected negative regulatory control by the energy responsive AMPK/mTOR pathway on the unfolded protein response (UPR) which is geared toward responding to ER stress by selectively allowing reparative proteins such as GRP 78 to be synthesized via initiation of translation thru *eIF2α*. Our findings suggest that due to the energy stress induced by this combinative treatment, mTOR is inhibited which results in blockage of GRP 78, a protein normally synthesized in response to ER stress. The significance of these findings are discussed in terms of an overall treatment strategy that takes advantage of windows of selectivity that naturally exist in most cancer cells due to their overall condition of increased stress as well as increased glucose uptake and metabolism.

## RESULTS

### Low concentration of 2-DG combined with FF induces necrotic and apoptotic cell death in different tumor cell lines under normoxia

**Figure 1:**
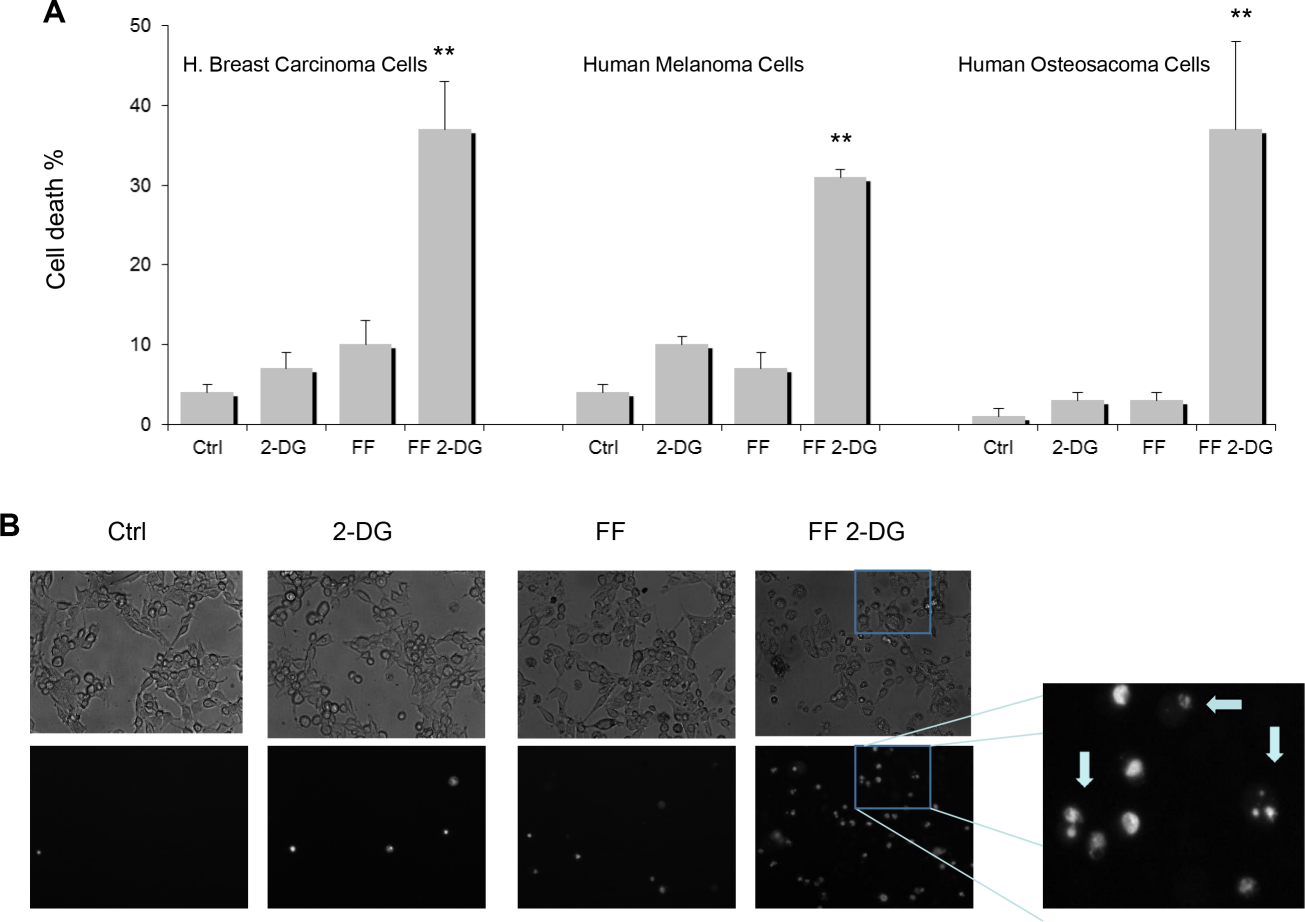
Synergistic cytotoxicity and different forms of cell death when combining FF and 2-DG at clinically achievable concentrations in tumor cell lines (**A**) Human breast carcinoma (SKBR 3), melanoma (NM2C5) and osteosarcoma (143B) cells were treated with FF (40 μM) and 2-DG (2 mM) either alone or in combination for 48 h followed by cell death analysis (**p < 0.01 values were determined compared to controls); (**B**) NM2C5 cells were treated for 24 h cells at 40 μM FF, 2 mM 2-DG or a combination of both. Morphological analysis of dead cells in the form of necrosis (i.e. faintly stained nuclear “ghosts,” indistinct vacuolated cytoplasm which appear light due to ruptured plasma membranes) and apoptosis, (i.e. cells in early stage of apoptosis show aggregated chromatin abutting the nuclear membrane, and condensed, basophilic cytoplasm) the latter indicated by arrows, was performed using DAPI and fluorescent microscopy

In order to test our hypothesis that exogenous ER stress could render tumor cells sensitive to FF, human breast carcinoma, melanoma and osteosarcoma cell lines were co-treated with a clinically achievable, non-toxic low concentration of 2-DG [11]. In Figure 1A it can be seen that 40 μM of FF (also at a non-toxic and clinically achievable dose) when combined with 2 mM of 2-DG leads to cell death in all three tumor cell lines. Using morphological analyses we find that 40 μM of FF + 2 mM of 2-DG induces cell death in the form of both necrosis (i.e. faintly stained nuclear “ghosts,” indistinct vacuolated cytoplasm which appear light due to ruptured plasma membranes) and apoptosis (i.e. cells in early stage of apoptosis show aggregated chromatin abutting the nuclear membrane, and condensed, basophilic cytoplasm) (Figure 1B). As illustrated in the morphological studies, the absence of toxicity with either 2 mM of 2-DG or 40 μMof FF alone, and synergistic increases in overall cell death at 24 h when the two are combined, confirm the toxicity results using the vi-cell viability assay as presented in Figure 1A.

### Metabolic effects of FF and 2-DG in tumor cells

**Figure 2:**
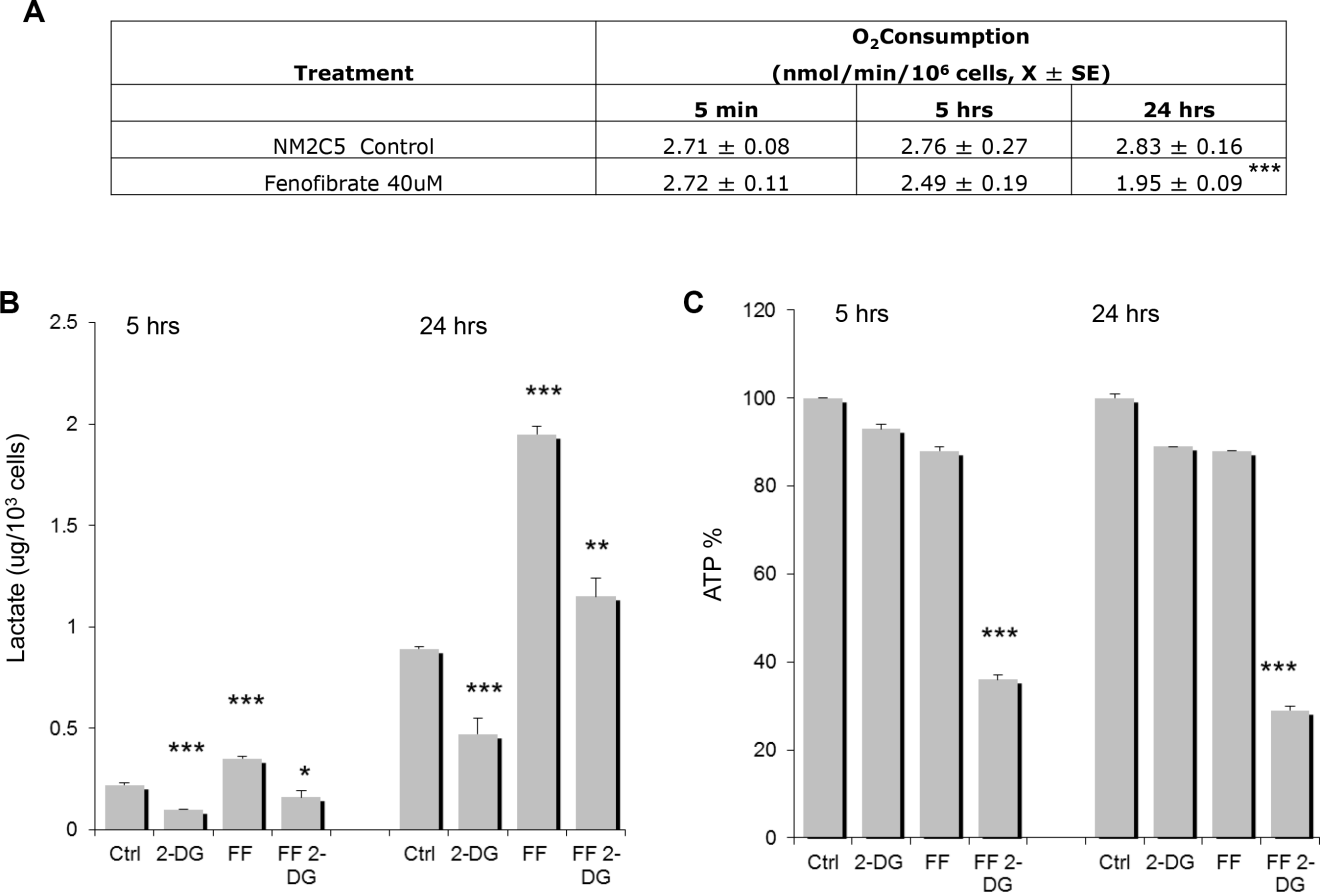
Oxygen consumption, lactate and ATP levels in cells treated with FF or 2-DG alone or in combination (**A**) Human melanoma NM2C5 cells were treated with FF (40 μM) and oxygen consumption was measured after 5 min, 5 h and 24 h of drug exposure (***p < 0.001, compared to controls); (**B**) FF (40 uM) or 2-DG (2 mM) or in combination were used to treat NM2C5 cells. Lactate levels in the medium were measured after 5 and 24 h of drug exposure and *p* values were *p < 0.05, **p < 0.01 and ***p < 0.001 as compared to controls: (**C**) FF (40 uM) or 2-DG (2 mM) alone or in combination were used to treat NM2C5 cells. Intracellular ATP levels were measured after 5 and 24 h of drug exposure and *p* values were ***p < 0.001 as compared to controls.

The effects of FF on respiration in isolated mitochondria were previously reported to be rapid and due to direct inhibition of the mitochondrial NADH:ubiquinone oxidoreductase (complex I) whereas in intact skeletal (soleus) muscle strips decreased respiration was delayed and recorded only after 24 h of treatment [10]. In the intact melanoma cell line NM2C5 tested, the effects of 40 μM of FF on respiration as measured by oxygen consumption could not be detected at early time points (5 min) but at later times (5 h) and (24 h) modest reductive effects were observed (Figure 2A). This result led us to investigate the ability of FF to convert NM2C5 metabolism from aerobic to anaerobic. It is well established that when mitochondrial function is inhibited, cells increase glycolysis and are forced to rely on this energy producing pathway for survival. Under these conditions pyruvate can no longer be efficiently oxidized by mitochondria, which results in a significant increase in lactate. Figure 2B illustrates that at an early time point (5 h), 40 μM of FF induces a 50% increase in lactate which increases further at 24 h (100%). Moreover, as expected, 2 mM of 2-DG alone lowers lactate about 50% at both time points, and when combined with 40 μM of FF, lactate levels stimulated by 40 μM of FF alone are similarly decreased. These results indicate that the low concentration of 2-DG (2 mM) used in these experiments is sufficient to inhibit glycolysis, at least in part, and that the clinically used concentration of 40 μM of FF-induced increase in lactate at 5 h is either due to FF's modest effects on mitochondrial oxygen consumption, or by another unknown mechanism.

### FF when combined with 2-DG lowers ATP levels and inhibits autophagy

Previously, it was shown that under anaerobiosis, cells treated with 2-DG or other glycolytic inhibitors at concentrations high enough to severely lower ATP, leads to cell death [12]. At 5 h, when 40 μM of FF is combined with 2-DG, ATP is lowered by 64% of control which is even more reduced at 24 h (71%) (Figure 2C). This severe drop in ATP leading to significant energy stress correlates with the necrotic form of cell death we observe when the various tumor cell lines as shown in Figure 1 are treated with 2 mM of 2-DG + 40 μM of FF.

**Figure 3:**
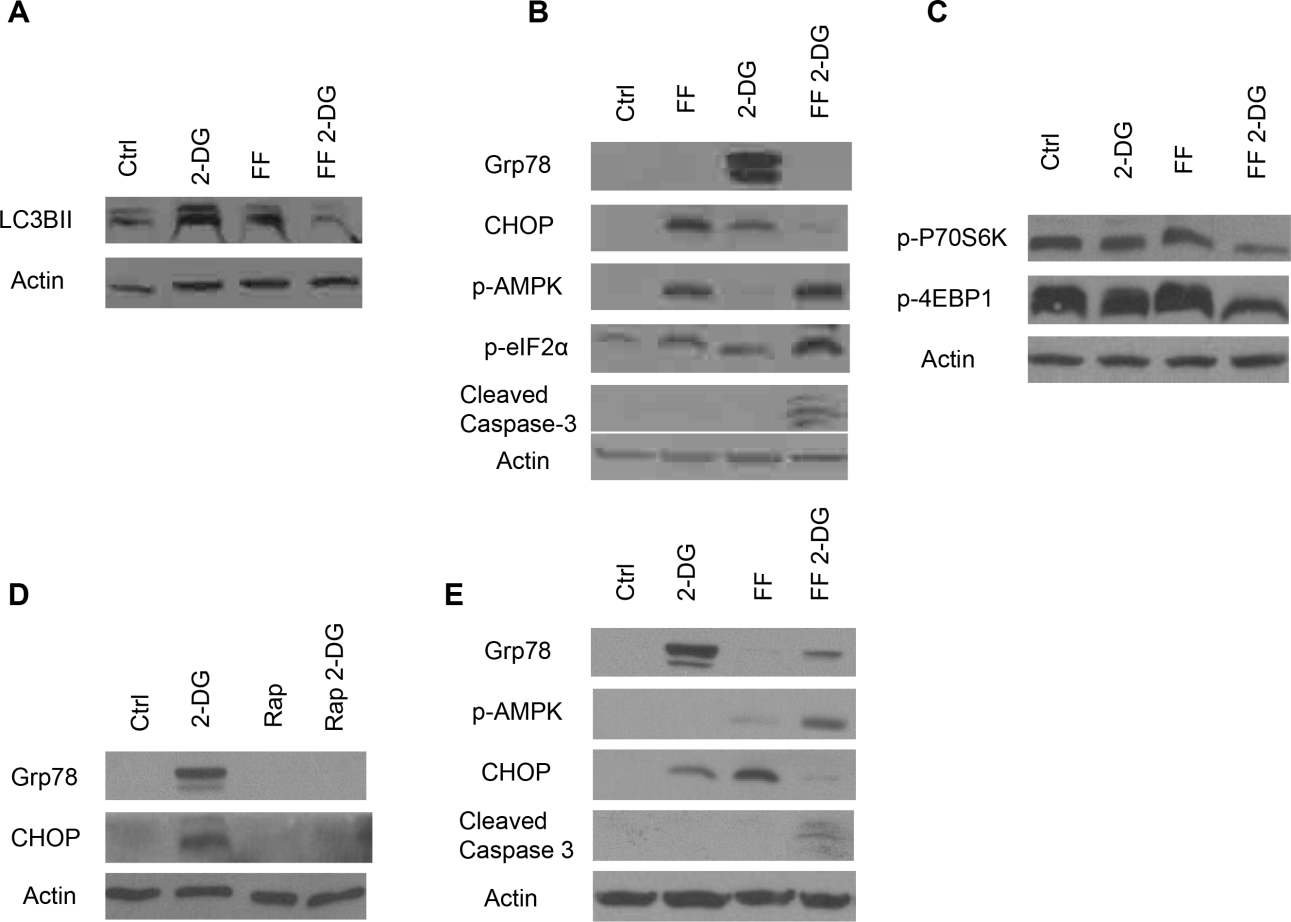
Autophagy activation, energy and ER stress induction in cells treated with FF or 2-DG alone or in combination FF (40 uM), 2-DG (2 mM), Rapamycin (Rap) (0.1 ug/ml) alone or combination were used to treat NM2C5 cells for 24 h and western blot analysis was performed to detect protein levels of: (**A**) LC3B II (as an indicator of autophagy activation); (**B**) Grp78 and CHOP (indicating activation of UPR) and p-eIF2α (indicating ER stress as well as other possibilities, please see text), p-AMPK (energy stress) and cleaved caspase 3 (apoptosis); (**C**) p-P70s6k and p-4EBP1 (indicative of mTOR activity); (**D**) Grp78 and CHOP. β-Actin was used as a loading control in each; (**E**) Human osteosarcoma 143b cell line treated with either 2-DG, FF, or both, for 24 h and western blot analysis was performed to detect protein levels of Grp78, CHOP, p-AMPK and cleaved caspase 3. Representative blots of one of at least two independent experiments are shown.

In response to 2-DG induced ER stress, under aerobic conditions, activation of autophagy acts as a protective mechanism against cell death [13]. However, when 2-DG (at higher concentrations than that used here) was combined with the mitochondrial inhibitor, oligomycin, in a pancreatic cell line (1420), ATP levels dropped severely > 80% and autophagy was observed to be blocked leading to cell death [13]. Similarly, here we find that 2 mM of 2-DG induced activation of autophagy as indicated by increased levels of LC3BII, is lowered when cells are co-treated with 40 μM of FF (Figure 3A). These data suggest that the lowering of ATP leading to a reduced protective effect of autophagy when 2 mM of 2-DG is combined with 40 μM of FF, may also, at least in part, be contributing to cell death.

### Combining FF with 2-DG increases p-eIF2α, p-AMPK and caspase 3 cleavage, but attenuates GRP 78 and CHOP

It has been repeatedly shown, that as an inhibitor of N-linked glycosylation, 2-DG increases ER stress and the subsequent unfolded protein response (UPR) markers GRP 78, and CHOP [3, 5, 6]. Moreover, when this type of stress is too severe, prolonged, and not alleviated by the UPR mechanism and/or autophagy, the cell undergoes UPR-mediated apoptosis. Recently, we reported that the UPR marker, GRP 78 induced by low concentration of 2-DG in human pancreatic tumor cells treated under aerobic conditions, was inhibited under anaerobic conditions (co-treated with oligomycin) [13]. Similarly, here we find that when melanoma cells as well as human osteosarcoma cells are co-treated with 2 mM of 2-DG and 40 μM of FF, GRP78 is attenuated (Figure 3B, 3D), further confirming that 40 μM of FF is converting cells from aerobic to anaerobic metabolism. Additionally, CHOP, another protein up-regulated by the ER stress/UPR pathway, is also reduced by the combination of 2 mM of 2-DG and 40 μM of FF. However, phosphorylation of *eIF2α*, indicative of ER stress, as well as the energy stress sensor pAMPK, are both found to be increased (Figure 3B). The latter data led us to question whether down-regulation of GRP 78 by the combination of 40 μM of FF + 2 mM of 2-DG could result from the markedly lowered levels of ATP detected (Figure 2C), leading to increased levels of p-AMPK and resulting in attenuation of mTOR (as measured by reduced phosphorylation of its downstream targets (P70S6K and 4EBP1) (Figure 3C).

To directly test the role of mTOR in lowering GRP 78, melanoma cells were co-treated with 2-DG and rapamycin. Figure 3D illustrates that the GRP 78 induced by 2 mM of 2-DG alone is completely inhibited when cells are co-treated with rapamycin although there is no apparent cell toxicity. This result suggests that under conditions of energy stress (lowered ATP), components of the UPR required to respond to ER stress i.e. GRP 78 and CHOP are compromised. As noted above, the increased levels of *p-eIF2α* detected under these conditions could be indicative of greater ER stress, which may result from the blockage of mTOR-mediated protein translation of GRP 78, required for protein folding.

**Figure 4:**
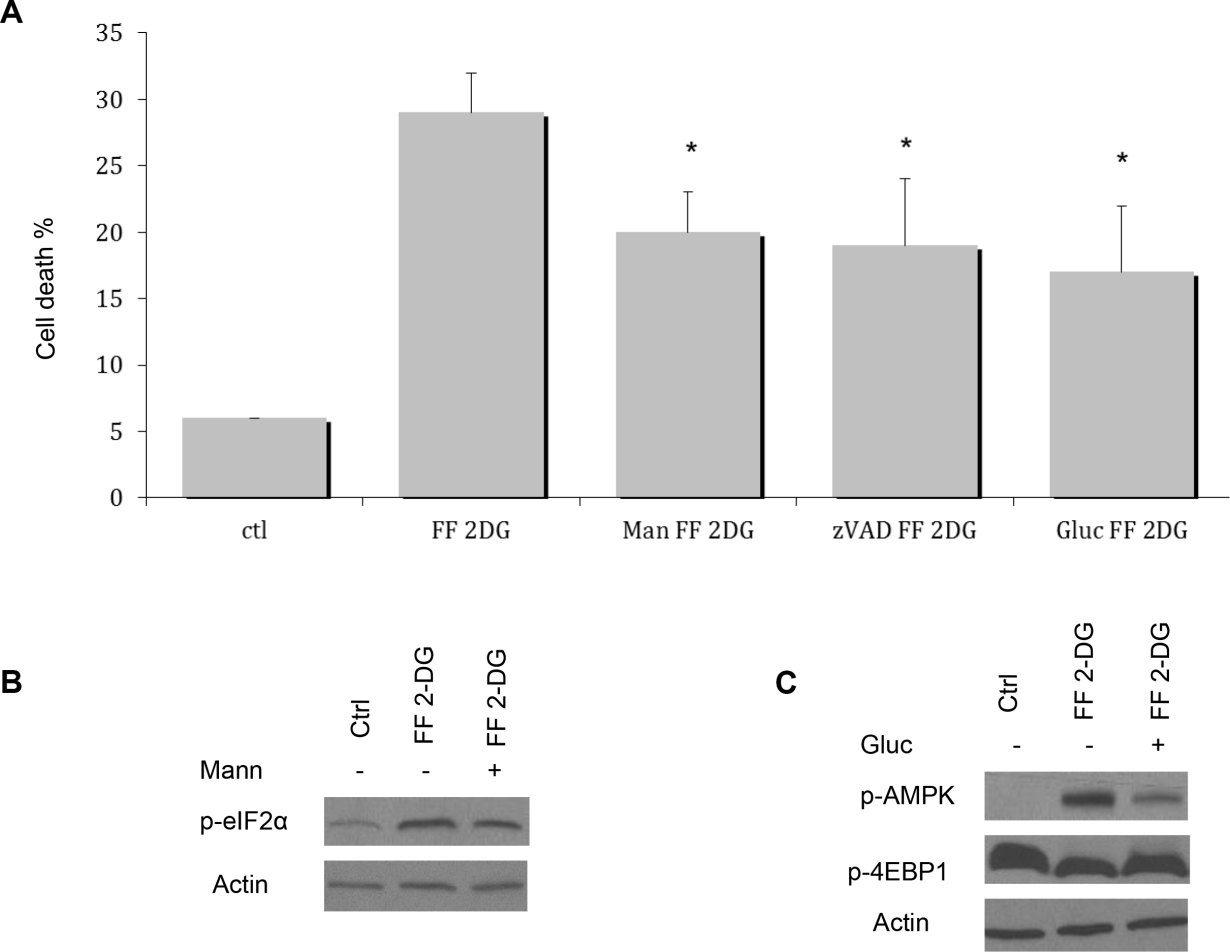
Effects of exogenous mannose or glucose on cell death induced by the combination of FF and 2-DG (**A**) Human melanoma NM2C5 cells were treated with 1 mM of mannose (Mann), 20 uM of Z-VAD-FMK (zVAD) or 1 mM of glucose (Gluc) in the presence of 40 μM FF, 2 mM 2-DG or combined, for 48 h followed by cell death analysis and *p* values were *p < 0.05 as compared to controls; (**B**) NM2C5 cells were treated with 1 mM of Mann in the presence of 40 μM of FF and 2 mM of 2-DG for 24 h. Western blot was performed to detect the level of p-eIF2a protein; (**C**) NM2C5 cells were treated with 1 mM of Gluc in the presence of 40 μM of FF and 2 mM of 2-DG for 24 h. Western blot was performed to detect levels of p-AMPK and p-4EBP1 proteins. β-Actin was used as a loading control. Representative blots of one of at least two independent experiments are shown.

In order to test the contribution of 2 mM of 2-DG induced ER stress to cell death when combined with 40 μM of FF, we co-treated NM2C5 cells with 1 mM of mannose. It had previously been reported that 2-DG, as an analog of mannose, interferes with the synthesis of oligosaccharides required for N-linked glycosylation [2, 3] which leads to ER stress [3, 4]. On the other hand, as an analog of glucose, 2-DG inhibits glycolysis which can result in energy stress especially when cells are treated under anaerobic conditions [4]. By adding low concentration of mannose to 2-DG treated cells, we developed a means of determining whether 2-DG toxicity is due to either ER or energy stress. Depending on the percent of 2-DG induced toxicity that is reversible by mannose, the contribution of ER stress to cell death can be assessed. In two select carcinoma cell lines that we reported to be unusually sensitive to low concentration of 2-DG under aerobic conditions, mannose was able to rescue 100% of cell death which was accompanied by marked lowering of ER stress markers [3]. Here we find that although 1 mM of exogenous mannose rescues cell death and concomitantly lowers ER stress in cells treated with 2 mM of 2-DG + 40 μM of FF, the reversal effects are only partial, indicating that in addition to ER stress other mechanisms must be contributing to its toxicity (Figure 4A and 4B). Moreover, our results with the pan-caspase inhibitor (Z-VAD-FMK), used in combination with 2 mM of 2-DG and 40 μM of FF show approximately the same amount (30%) of protection that adding 1mM of exogenous mannose (Figure 4A). Furthermore, we find the following which supports our contention that energy stress which leads to necrosis, significantly contributes to the mechanism of cell death: (a) adding 1 mM of glucose partially rescues the toxic effects of this combination with consequent AMPK reduction and increased phosphorylation of 4EPB1 (indicative of increased mTOR function) (Figure 4C); (b) 2- fluorodeoxy-D-glucose (2-FDG) which we previously demonstrated to be a more potent inhibitor of glycolysis but less potent inducer of ER stress than 2-DG [14] was more effective in combination with 40 μM of FF in killing the tumor cell lines tested in this study (data not shown). Overall, these results support a mechanism of tumor cell death by 40 μM of FF + 2 mM of 2-DG involving attenuation of the UPR by mTOR adding to the burden of both energy and ER stress induced by this combination of drugs.

### The combination of 2-DG+ FF increases Noxa and downregulates Mcl-1 expression

**Figure 5:**
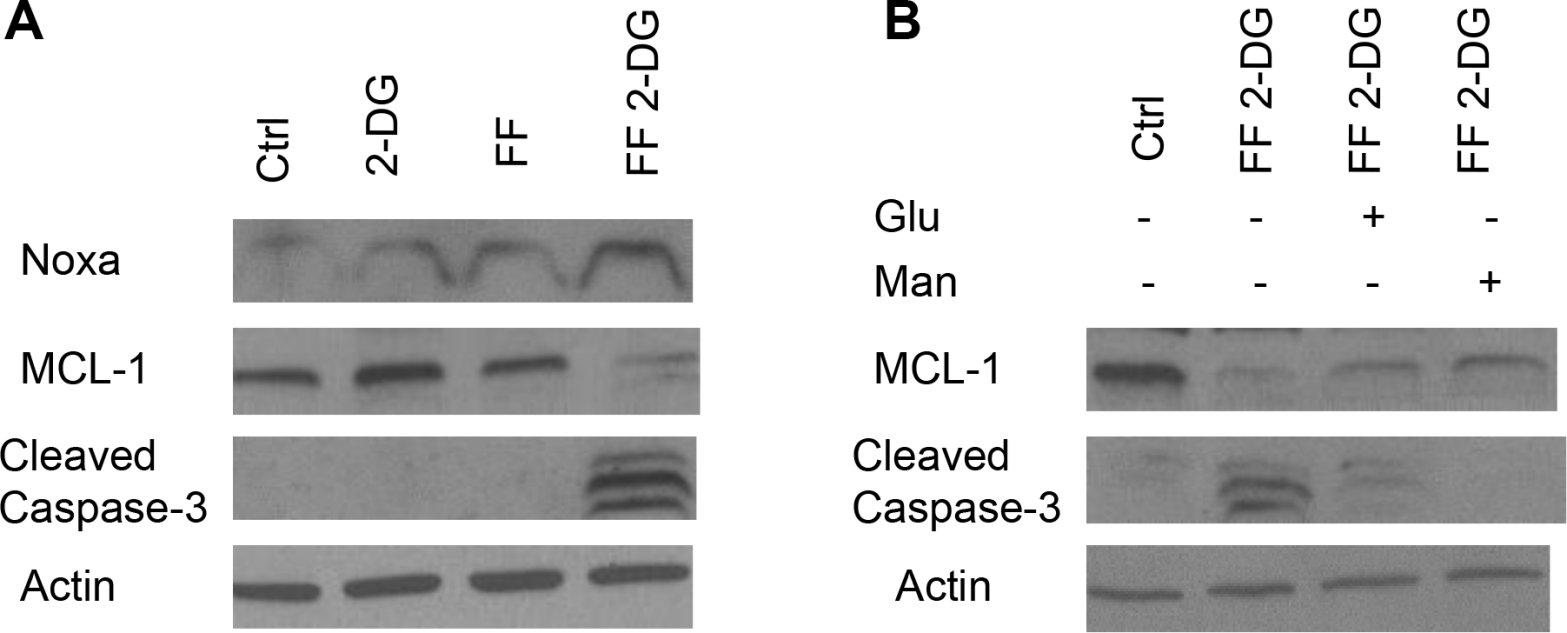
Effects of 2-DG + FF on apoptotic proteins Noxa and Mcl-1 (**A**) FF (40 uM), 2-DG (2 mM) alone or in combination were used to treat NM2C5 cells for 24 h. Western blot analysis was performed to detect levels of Noxa, Mcl-1 and cleaved caspase 3 proteins; (**B**) NM2C5 cells were treated with 1 mM of Man or Glu in the presence of FF (40 μM) combined with 2-DG (2 mM) for 24 h. Western blot was performed to detect levels of Mcl-1 and cleaved caspase 3 proteins. β-Actin was used as a loading control. Representative blots of one of at least two independent experiments are shown.

**Figure 6:**
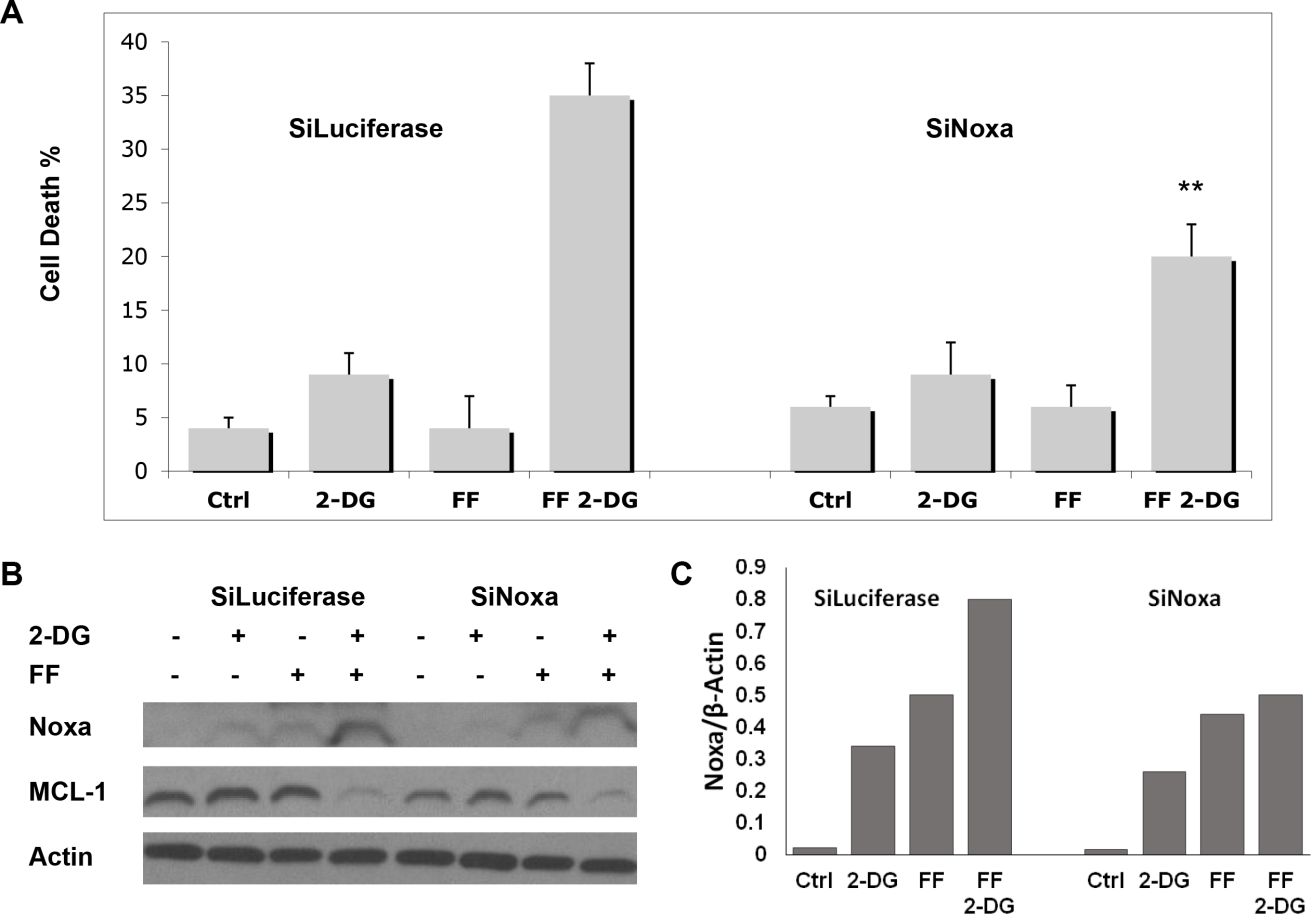
Effects of Knock-down of Noxa by SiRNA on cell death induced by the combination of FF and 2-DG (**A**) NM2C5 cells were incubated with siRNA against either luciferase (siLuc, as negative control) or Noxa (siNoxa) for 24 h before addition of 40 μM of FF, 2 mM of 2-DG or a combination of both as indicated. Following 72 h of treatment, cell death analysis was performed and *P* values **p < 0.01, were compared to FF combined with 2-DG treatment in the control (Siluc) group; (**B**) Western blot analysis of Noxa protein levels in NM2C5 cells treated as described in (A); (**C**) Quantification (ratio of Noxa/β-Actin) of Noxa levels presented in Figure 6 (B). β-Actin was used as a loading control. Representative blots of one of at least two independent experiments are shown.

As noted above, Ramírez-Peinado et al. [5] reported that 2-DG is toxic to a number of rhabdomyosarcoma cell lines when treated under normoxia which correlated with up-regulation of the pro-apoptotic BH3-only protein Noxa and consequent down-regulation of its anti-apoptotic protein target, Mcl-1. This result prompted us to examine the effects of combining FF + 2-DG on this pair of pro and anti -apoptotic proteins. Figure 5A illustrates that the combination of 40 μM of FF + 2 mM of 2-DG results in increased Noxa and decreased levels of Mcl-1, as well as increased caspase 3 cleavage, indicative of apoptotic-mediated cell death. In order to determine whether changes in levels of these apoptotic proteins were involved with the toxicity induced, cells treated with 2 mM of 2-DG + 40 μM of FF were co-treated with siRNA specific to Noxa. In Figure 6A it can be seen that significant but not complete protection from cell death was achieved with this treatment although levels of Mcl-1 did not appear to recover (Figure 6B). Thus, the 2 mM of 2-DG + 40 μM of FF-induced reduction in Mcl-1 levels is more likely mediated by translational inhibition (mTOR and or eIF2α) due to metabolic and/or ER stress, and not by upregulation of Noxa. In support of this interpretation, are data which demonstrate that when the toxicity induced by 40 μM of FF + 2 mM of 2-DG was rescued by either 1 mM of mannose or glucose (Figure 4A), the functionality of eIF2α and mTOR (4EBP1) were found to be recovered (Figure 4B and 4C), corresponding with increases in Mcl-1. These results indicate that the levels of Mcl-1 are regulated by both ER and energy stress since glucose as well as mannose partially recovered the amounts of this anti-apoptotic protein decreased by 2-DG + FF (Figure 5B) which agrees with the partial cell death rescue shown in Figure 4A. Overall, these data demonstrate that although we cannot detect lowering of Mcl-1 by 2 mM of 2-DG or 40 μM of FF alone in the melanoma cell line studied, combining the two, results in marked reduction of this critical anti-apoptotic protein which leads, at least in part, to cell death.

### Combining FF with 2-DG decreases tumor volume and increases caspase 3 in a human melanoma xenograft model

**Figure 7:**
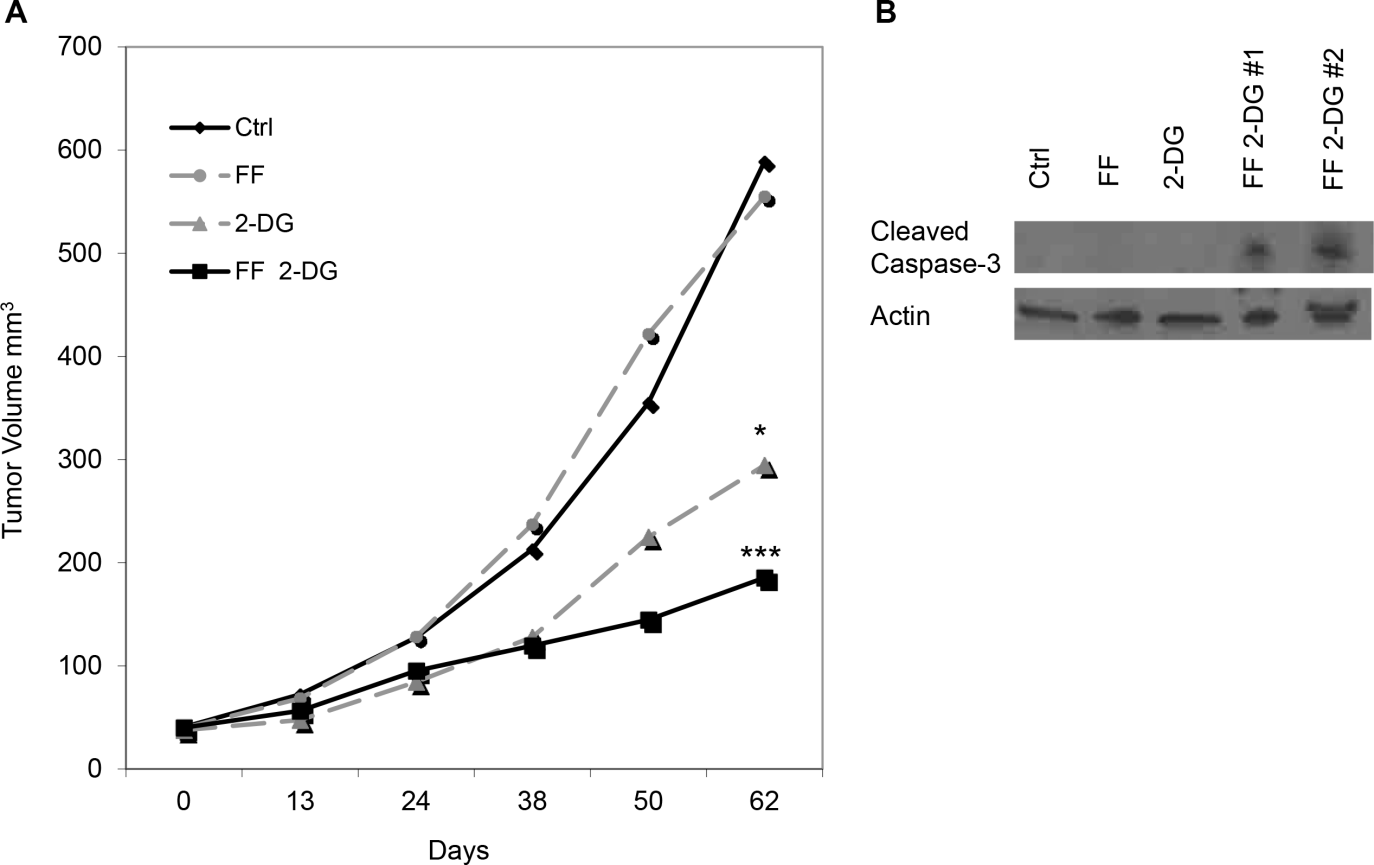
Effect of 2-DG and FF, alone and in combination, on growth of human melanoma xenografts in nude mice (**A**) Mice (*n* = 8/group) were treated either FF only (100 mg/kg/day, oral gavage daily), 2-DG (41 ug/ml/hr, ALZET pumps subcutaneously implanted) only, or a combination of FF (100 mg/kg/day) and 2-DG (41 ug/ml/hr). Mice in the control group had saline oral gavage and ALZET pump delivery. Tumor volumes were measured weekly after treatment. Representative tumor volumes of one of two independent experiments are shown. *P* values *p < 0.05 and ***p < 0.001 are as compared to saline control and *p* = 0.116 is FF + 2-DG as compared to 2-DG alone. (**B**) Western blot was performed to detect level of cleaved caspase 3 in the samples of tumor tissue. β-Actin was used as a loading control.

The results from our Phase I clinical trial showed that although 2-DG was well-tolerated in patients, by drinking it once per day an insulin response was induced [11]. Although it was not measured, it can be assumed that 2-DG, similar to glucose, under these conditions would be redirected to muscle and fat tissue and away from the tumor. Additionally, at a high enough level, 2-DG like glucose could be adsorbed by the liver thereby reducing its tumor concentration. Based on these considerations we performed experiments in a human melanoma xenograft mouse model. Mice received either FF only (100 mg/kg/day, oral gavage daily), 2-DG (41 ug/ml/hr, ALZET pumps subcutaneously implanted) only, or a combination of FF (100 mg/kg/day) and 2-DG (41 ug/ml/hr). Mice in control group had saline oral gavage and ALZET pump delivery. Our results indicate that the combination of FF (100 mg/kg/day) +2-DG (41 μg/ml/hr) is safe and effective in lowering tumor burden and that slow-release pumping of 2-DG (41 μg/ml/hr) appears to give the best anti-tumor results when combined with 100 mg/kg/day of FF (Figure 7A). Moreover, the total dose per week, 462 mg/kg, delivered by this method is more than 3 times lower than the total dose previously shown to have activity when animals were treated by IP injection 3 times per week [15]. Additionally, the increased caspase III in the samples of tumor tissue recovered from 2-DG (41 μg/ml/hr) + FF (100 mg/kg/day) treated animals (Figure 7B), correlates with our *in vitro* findings. To our knowledge this is the first report showing that 2-DG can be effectively delivered in this manner, as well as safely combined with FF *in vivo*, warranting further consideration as 2-DG continues to be developed for patient use.

## DISCUSSION

Although the discovery of oncogenic pathways has led to improvements in cancer treatment, due to inherent heterogeneity in cancer evolution, redundancy in signaling pathways and mechanisms of resistance, limited success has been achieved [16]. Thus, a more comprehensive approach where a universal vulnerability of cancers can be exploited is needed. Increased glucose metabolism offers such a target since glucose is not only a vital energy source but also supplies the carbons required for cell replication and growth [17, 18]. 2-DG seems to be well-suited to take advantage of this natural window of selectivity as it has been shown to be selectively taken up by tumor vs normal cells. Additionally, by simultaneously inducing energy and ER stress and thereby further adding to increased stress that malignant cells are already under, 2-DG is able to exploit another natural window of selectivity that exists between cancer and normal cells.

When tumor cells are treated under aerobic conditions at high enough concentrations of 2-DG that completely block glycolysis, they are able to survive by using other energy sources i.e. fats and proteins to generate ATP via oxidative phosphorylation pathway. However, under hypoxia or anaerobiosis, cells are forced to rely on glycolysis for survival and when it is shut down by 2-DG, precipitous drops in ATP levels, lead to energy stress great enough to induce cell death [12]. It should be noted that although high concentration of 2-DG also lowers ATP under normoxic conditions, the levels of reduction are in a range of 30 to 40%, whereas under hypoxia they are reduced by 70 to 90% lower than controls [12]. Thus, under aerobic conditions the amount of energy stress induced by 2-DG's inhibitory effects on glycolysis even at high dose does not appear to be sufficient to cause toxicity in most carcinoma cell types tested [19]. Moreover, since 2-DG as an analog of mannose interferes with the growing oligosaccharide chain required for proper folding of N-linked glycosylated proteins, it also induces ER stress [2, 3]. We as well as others have shown that under normoxic conditions a variety of different cancer cell types i.e. a few select carcinomas, rhabdomyosarcomas, acute lymphoblastic leukemias and others, treated with 2-DG at low concentration undergo cell death mainly due to ER stress/UPR mediated apoptosis [3, 5, 6]. Thus, the fortuitous nature of 2-DG in inducing both kinds of stress simultaneously, coupled with its selective uptake driven by oncogene-directed increased glucose metabolism, make this glucose/mannose analog unique as an anti-tumor agent in its widespread application.

Based on our findings that multiple myeloma cells which are already under a heightened state of ER stress, are particularly vulnerable to agents that inhibit mitochondrial function, led us to question whether cancer cells in general could be made to be “multiple myeloma like” in their sensitivity to FF by inducing ER stress exogenously using 2-DG. As determined in dose response experiments (Supplementary Figure 1) and as shown in Figure 1, at concentrations of 2-DG or FF that are non-toxic when applied alone, combining them induces cell death in the form of both necrosis and apoptosis in three different tumor cell types. This result led us to determine whether the effects of FF on mitochondrial function are responsible for rendering cells vulnerable to 2-DG's activity as a glycolytic inhibitor. In this regard, at an early time point (5 h) we could only detect a modest effect on oxygen consumption by FF (Figure 2A) and yet we did observe a statistically significant increase in lactate production (Figure 2B) indicating that glycolysis is also increased. This result suggests that FF induces lactate production via a mechanism independent of inhibiting oxygen consumption which may still be mediated via mitochondrial effects.

Most importantly, the combination of FF and 2-DG is found to lower ATP significantly (Figure 2C) suggesting that energy stress due to the glycolytic inhibitory activity of 2-DG combined with effects of FF on mitochondria at least in part contributes to the cytotoxicity of this combination of drugs. Additionally, when 1 mM of glucose is added to the medium, energy stress is reduced as reflected in lowered pAMPK signal and increased phosphorylated 4EBP1 indicative of mTOR function which also results in partial recovery from toxicity and interestingly increases in Mcl-1 levels, further reinforcing the idea that both forms of stress are contributing to the different types of cell death. Moreover, our data which show that mannose or the pan-caspase inhibitor Z-VAD only reverses 30 to 35% of the cell death further supports the contention that energy as well as ER stress are combining to induce cell death. Additionally, our morphological results of the types of cell death induced by this combination support our interpretation that at least two different forms of stress are involved in inducing cell death. Furthermore the partial reversal of toxicity that is observed when mannose is added to 2-DG and FF, which correlates with less ER stress as measured by decreased eIF2α phosphorylation, as well as with increased Mcl-1 levels, further supports the interpretation that ER stress-mediated apoptosis contributes to cell death. It should be noted that our data do not exclude the possibility that other forms of stress such as oxidative may also be involved with 2-DG + FF –induced cell death as has been shown for 2-DG alone at higher concentrations than those used here [20, 21]. Further investigation assaying ROS as well as blocking it will be required to assess the role that oxidative stress plays, if any in the cytotoxicity induced by 2-DG+ FF.

An increase in the level of GRP 78, the folding protein central to signaling the UPR to respond to, and correct unfolded proteins, is routinely used as a marker of ER stress. As we reported previously, 2-DG-induced increase in GRP 78 is attenuated under anaerobic conditions [13]. We observed a similar result when cells were treated with FF + 2-DG which led us to question whether the energy stress (lowered ATP > 70%) generated by this combination, leading to increased pAMPK and resulting in down-regulation of mTOR (as measured by reduction in phosphorylation of its downstream targets P70S6K and 4EPB1) was responsible for lowering GRP 78. In order to directly address this question, cells were co-treated with 2-DG and the mTOR, inhibitor, rapamycin. As shown in Figure 4C, the robust increase in GRP 78 in response to 2-DG was significantly reduced by the addition of rapamycin. Thus, a possible mechanism to explain the similar result observed when cells are treated with FF + 2-DG is that under conditions where energy stress is high enough to block protein synthesis directed by mTOR, the translation of components of the UPR required to respond to ER stress is attenuated. Moreover, under these conditions increased phosphorylation of eIF2α is detected indicating additional ER stress. We speculate that this could be due to insufficient GRP 78 required for an appropriate response to the initial amount of mis-folded proteins induced by 2-DG. This possibility remains speculative since it is well known that mechanisms other than ER stress regulate eIF2α phosphorylation i.e. viral infection, nutrient and heme-deprivation, [23]. In addition to these other mechanisms it has recently been reported that RHEB GTPase which stimulates mTOR activity also phosphorylates eIF2α attenuating protein translation [24]. Thus, when mTOR is shut down either by FF + 2-DG or rapamycin, RHEB may be playing a role in the increased phosphorylation we detect in eIF2α which may not necessarily reflect increased ER stress. Interestingly, CHOP is also found to be reduced by 2-DG + FF treatment which would be expected to reduce ER-stress mediated cell death. The fact that apoptosis does occur, indicates that either there is enough CHOP signal to initiate cell death and/or other pathways are responsible. Overall, these experiments reveal that mTOR, a translation regulator that has evolved to respond to energy and nutrient stress, can interfere with mechanisms designed to respond to ER stress. The fact that under ER stress p-eIF2α does not inhibit the translation of GRP 78 and several other proteins required for reparative and survival responses, but under energy stress mTOR does, may at least in part explain the cell death observed in cells treated with the combination of 2-DG and FF.

Among the apoptotic proteins required to respond to ER stress, Mcl-1 appears to be particularly important for cell survival [25]. Thus, our findings that FF + 2-DG significantly lower Mcl-1 levels suggest that this protein is involved with the mechanism of survival to this treatment. The reduction in Mcl-1 levels can be mediated by either direct translational (mTOR and or Eif2 alpha) attenuation of the protein or by binding to Noxa, which then is degraded by the ubiquitin system. Although knock-down of Noxa was able to reverse 30% of the 2DG+FF induced toxicity (6a) surprisingly Mcl-1 levels did not appear to change (6b). This result suggests that Noxa may be mediating 2DG + FF -induced toxicity independently of its effects on Mcl-1. Overall, treatment with 2DG and FF is found to result in Noxa up-regulation and Mcl-1 down-regulation which appears to occur through distinct pathways culminating in induction of apoptosis.

In summary, the results presented here indicate that by increasing energy as well as ER stress in tumor cells that are already under more stress than normal cells, the combination of FF and 2-DG may provide a universal means by which different cancer types in general may be targeted. Moreover, 2-DG as a dual energy and ER stress inducing agent, seems particularly well-suited to take advantage of this natural window of selectivity that heightened tumor stress provides. In addition, increased glucose metabolism, which has been demonstrated by numerous PET scans to be a feature of most tumors and more recently shown to be driven by major oncogenes [26], presents a universal target that can be naturally exploited by 2-DG. The fact that slow-release pump seems to be an effective way to deliver 2-DG and that combined with oral FF shows significant anti-tumor activity *in vivo* provides promise for developing this combination clinically and others that combine 2-DG with agents that act synergistically to selectively increase energy and ER stress to a level that is toxic to numerous tumor cell types.

## MATERIALS AND METHODS

### Cell culture

Human cell lines i.e. melanoma, NM2C5, osteosarcoma, 143B and breast adenocarcinoma, SKBR3 were purchased from American Type Culture Collection (Manassas, VA), and maintained in DMEM, 1 g/L of glucose supplemented with 10% fetal bovine serum and penicillin/streptomycin (Invitrogen, NY). Cells were grown under 5% CO2 at 37°C.

### Drugs and antibodies

2-Deoxy-glucose, mannose, D-(+)-Glucose, rapamycin, and fenofibrate (were purchased from Sigma-Aldrich (St. Louis, MO). General Caspase Inhibitor Z-VAD-FMK (FMK001) was from R&D Systems (MN). The following rabbit primary antibodies used were from Cell Signaling Technology (Danvers, MA): LC3B (2775 which preferentially detects LC3B-II), Grp78 (3177), CHOP (2895), pAMPKa (Thr172, 2535), p-p70S6K (Thr389, 9234), p-eIF2a (Ser51, 3597), p-4EBP1 (Thr37/46, 9459), Cleaved Caspase-3 (9664) and Mcl-1 (4572). Mouse anti-b-actin (A5441) primary antibody was from Sigma-Aldrich. Mouse anti-Noxa was from Calbiochem (OP180). Horseradish peroxidase-conjugated anti-rabbit (W4011) and anti-mouse (W4021) secondary antibody were purchased from Promega (Madison, WI).

### Cytotoxicity assay

Cells were seeded onto 24-well plates and cultured for 18–22 h. After drug exposure for 48 or 72 h, attached cells were removed by trypsinization and their respective culture media were collected and centrifuged as described previously (3). The pellets were then re-suspended in Hanks Balanced Salt Solution (HBSS) (Mediatech) and analyzed with Vi-Cell cell viability analyzer (Beckman Coulter) based on trypan blue exclusion. Results were shown as the percent dead cells out of the total counted. Data were the averages of triplicate samples +SD from one representative experiment out of at least three independent ones unless otherwise indicated.

### Fluorescent microscopy

Cells were seeded onto 24-well plates and cultured for 18–22 h to approximately 60% confluence. After 24 h of drug exposure, cells were stained with DAPI (0.5 μg/ml, a gift from Dr. Xiang-Xi Xu) and visualized immediately with the Nikon Eclipse TE2000 microscope (Nikon, Melville, NY) to analyze changes in nuclear condensation and fragmentation. Microphotographs of the center of each well were taken at 60X magnification with the aid of imaging-capture software (NIS-Elements from Nikon, Melville, NY). Cells shown were from one representative experiment out of at least three independent analyses.

### Oxygen consumption

Oxygen consumption was measured by placing 2 × 10^6^ cells into a closed chamber equipped with a Clark electrode (Hansatech, Cambridge, UK) as described previously [27]. Data were the averages of samples ± SE from three (5 min and 5 h) or six (24 h) independent experiments.

### Lactate assay

At 24 hr after seeding and grown cells were rinsed twice with PBS, fresh medium was added to the control and test cultures, at the indicated drug concentrations, and the cells were re-incubated in 5% CO2 for 5 or 24 h. Then, 0.5 ml of medium was removed from each culture and deproteinated by adding 1 ml of perchloric acid at 8% w/v, vortexing for 30 s, then placing this mixture in 4_ C for 5 min, and centrifuging at 1,500 g for 10 min. The supernate was centrifuged three times more. To determine lactic acid, 0.025 ml of this supernate was added to a reaction mixture containing 0.1 ml of lactic dehydrogenase (1,000 units/ml), 2 ml of glycine buffer (glycine, 0.6 mol/l, and hydrazine, pH 9.2), and 1.66 mg/ml NAD. Formation of NADH was measured with a Beckman DU 520 UV/VIS spectrophotometer at 340 nm, which directly corresponded to lactic acid levels as determined by a lactate standard curve. Readouts from control samples, normalized to cell number, were set at 100% and those from all the other samples were presented as percentages of controls. Samples were analyzed in triplicate.

### ATP quantification

Intracellular ATP levels were measured with the CellTiter-Glo Luminescent Cell Viability Assay (Promega, WI) according to the manufacturer's directions. Results were the averages of triplicate samples +/− SD from one representative experiment out of at least three independent ones. Short-time (5 h or 24 h) treatment was employed to minimize the toxicity and cell number loss caused by drug exposure.

### Western blot

Cells were seeded onto six-well plates and cultured for 18–22 h to reach approximately 70% confluence. Following drug exposure for the indicated times, cells were harvested and lysed with the lysis buffer (100 mM Tris–HCl at pH 7.4, 1% SDS, phosphatase inhibitor cocktail 2 and protease inhibitor cocktail from Sigma–Aldrich). Protein concentrations of each sample were determined using a Micro BCA Protein Assay Kit (Thermo Scientific) according to the manufacturer's directions, and equal amounts of proteins were loaded onto 4–15% Tris–HCl gradient gels (Bio-Rad). SDS–PAGE proteins were transferred onto a polyvinylidene fluoride (PVDF) membrane (Millipore), blocked with 5% milk and probed with corresponding primary antibodies overnight (except 1 h for actin). The membrane was washed and probed with secondary antibodies for 1 h and was then incubated with SuperSignal West Pico or Femto Chemiluminescent Substrate (Thermo Scientific) and signals were visualized on Blue Lite Autorad Films (ISCBio- Express). All primary antibodies were used at 1:1000 dilution except for actin (1:10000), and the secondary antibodies were used at 1:10000.

### siRNA transfection

Cells were seeded into 12 welled plates and cultured for 24 h to reach approximately 30% confluence using antibiotic-free media. Then, cells were transfected with either anti-Luc siRNA-1 (targeting luciferase) or Noxa siRNA purchased from Santa Cruz Biotechnology, INC (sc-37305). Twenty-four hours after transfection, cells were treated for the indicated times and collected for immunoblotting or cytotoxicity analyses. The lowest concentrations of siRNAs were determined by dose response experiments which produced the most efficient knockdown with minimal toxicity.

### Statistics

Statistical analyses were performed by two-tailed unpaired Student's *t*-test, and a *P* value less than 0.05 was considered significant.

### Mouse xenograft studies

All animal experiments were carried out in strict accordance with to the guidelines of the Association for Assessment and Accreditation of Laboratory Animal Care (AAALAC) International and the recommendations in the Care and Use of Laboratory Animals of the National Institute of Health. Eight-week-old female immunodeficient CD-1 nude mice (Charles River Laboratories, USA) were inoculated subcutaneously with 0.1 ml of tumor cells (2 × 10^7^ cells/ml in Hank's). Fourteen days later tumor-bearing mice were randomized into four groups of 8 mice each, each with comparable tumor size. FF was formulated in 1% methylcellulose and 1% Tween-80. 2-DG was in PBS. Mice received either FF only (100 mg/kg/day, oral gavage daily), 2-DG (41 ug/ml/hr, ALZET pumps subcutaneously implanted) only, or a combination of FF and 2-DG. Mice in control group had saline oral gavage and ALZET pump delivery. Tumor size was measured once weekly by electronic calipers and calculated tumor volume based on ellipsoid dimensions (width 2 × length × 0.5). All mice were maintained under specific pathogen-free conditions in accordance with guidelines set forth by AAALAC International. Implanting ALZET pumps: Mice were anesthetized by 62 mg of ketamine hydrochloride and 5 mg of Xylazine/kg i.p. The ALZET pumps were filled with 2-DG or saline following the manufacture's instruction of making a skin incision in the back of mouse. Inserting the pump filled with saline or 2-DG and close the incision with 2 or 3 wound clips.

## SUPPLEMENTARY MATERIALS FIGURE


