# Supplementary material for: Combining 2-deoxy-D-glucose with fenofibrate leads to tumor cell death mediated by simultaneous induction of energy and ER stress
Source: Oncotarget. 2016 May 10;7(24):36461–73. doi: 10.18632/oncotarget.9263 (PMC5095013; doi:10.18632/oncotarget.9263)
Supplement: Supplementary file 1 [file oncotarget-07-36461-s001.pdf]

Combining 2-deoxy-D-glucose with fenofibrate leads to tumor cell death mediated by simultaneous induction of energy and ER stress

Supplementary Materials

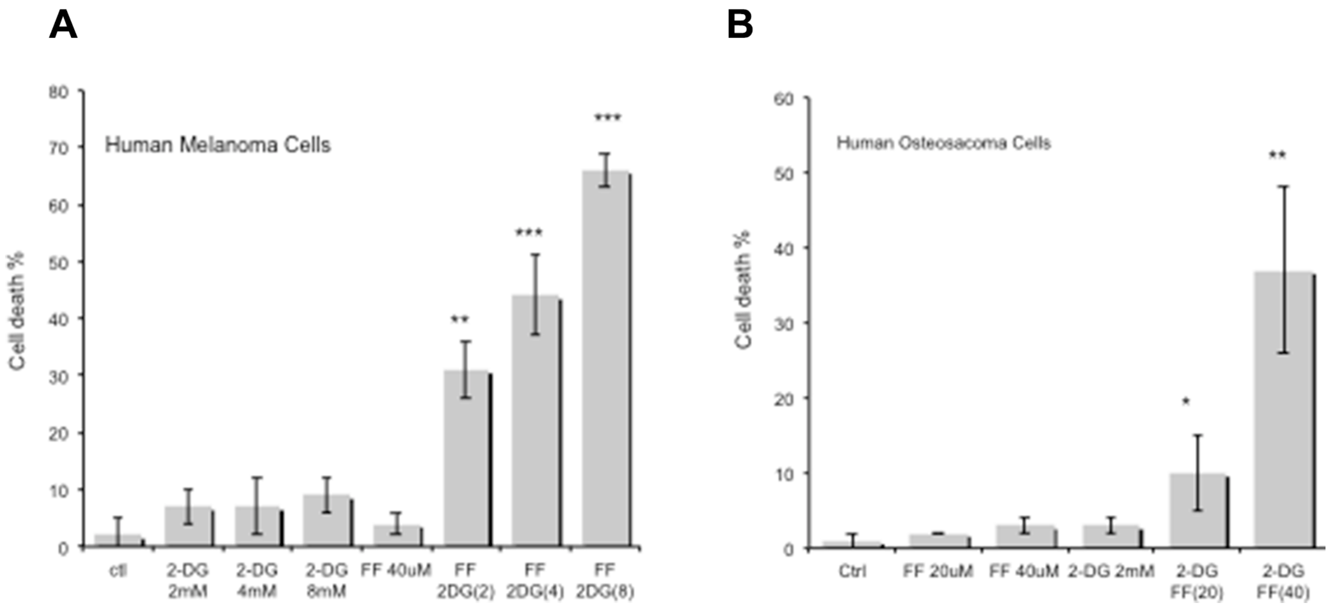

Supplementary Figure S1: Dose response of 2-DG and FF cytotoxicity in tumor cell lines. (**A**) Melanoma (NM2C5) and (**B**) osteosarcoma (143B) cells were treated with FF and 2-DG at increasing concentrations of either drug alone or in combination for 48 h followed by cell death analysis (**$p < 0.01$ values were determined compared to controls).
